# Presynaptic GABA_B_ Receptor Regulates Activity-Dependent Maturation and Patterning of Inhibitory Synapses through Dynamic Allocation of Synaptic Vesicles

**DOI:** 10.3389/fncel.2012.00057

**Published:** 2012-12-03

**Authors:** Yu Fu, Xiaoyun Wu, Jiangteng Lu, Z. Josh Huang

**Affiliations:** ^1^Cold Spring Harbor LaboratoryCold Spring Harbor, NY, USA; ^2^Program in Neuroscience, Stony Brook UniversityStony Brook, NY, USA

**Keywords:** GABAB receptor, synaptic vesicle dynamics, live cell imaging, actin polymerization, FRET, inhibitory synapses, activity-dependent development

## Abstract

Accumulating evidence indicate that GABA regulates activity-dependent development of inhibitory synapses in the vertebrate brain, but the underlying mechanisms remain unclear. Here we combined live imaging of cortical GABAergic axons with single cell genetic manipulation to dissect the role of presynaptic GABA_B_ receptors (GABA_B_Rs) in inhibitory synapse formation in mouse. Developing GABAergic axons form a significant number of transient boutons but only a subset was stabilized. Synaptic vesicles in these nascent boutons are often highly mobile in the course of tens of minutes. Activation of presynaptic GABA_B_Rs stabilized mobile vesicles in nascent boutons through the local enhancement of actin polymerization. Inactivation of GABA_B_Rs in developing basket interneurons resulted in aberrant pattern of bouton size distribution, reduced bouton density and reduced axon branching, as well as reduced frequency of miniature inhibitory currents in postsynaptic pyramidal neurons. These results suggest that GABA_B_Rs along developing inhibitory axons act as a local sensor of GABA release and promote presynaptic maturation through increased recruitment of mobile vesicle pools. Such release-dependent validation and maturation of nascent terminals is well suited to sculpt the pattern of synapse formation and distribution along axon branches.

## Introduction

Inhibitory synaptic innervation in the cerebral cortex is often characterized by specificity as well as robustness (Somogyi et al., 1998; Huang et al., 2007). For example, fast-spiking basket interneurons selectively contact the soma and proximal dendrite of pyramidal neurons, and a single basket cell innervates hundreds of pyramidal neurons with tens of clustered perisomatic synapses on each target (Tamas et al., 1997; Chattopadhyaya et al., 2007). Perisomatic inhibitory synapses contribute to the effective control of the output and synchrony of pyramidal neurons (Jonas et al., 2004; de la Prida et al., 2006; Cardin et al., 2009), but the underlying developmental mechanisms are largely unclear. At the developing glutamatergic synapses, dendritic and axonal filopodia actively search for and initiate contacts to pre- or post-synaptic counterpart within their reach, and glutamate signaling is crucial in the activity-dependent validation and maturation of excitatory synapses (Ahmari et al., 2000; Fischer et al., 2000; Friedman et al., 2000; Chang and De Camilli, 2001; Craig et al., 2006; Shen et al., 2006). On the other hand, inhibitory synapses often form in the absence of postsynaptic protrusions, thus GABAergic axons likely play a more active role in searching for and soliciting synaptic targets. For example, dendrite-targeting GABAergic synapses are formed exclusively at pre-existing axon-dendrite crossings without the involvement of dendritic protrusions (Wierenga et al., 2008).

In the developing neocortex which consists of diverse and intermingled cell types, a basket interneuron axon explores a highly complex cellular milieu with myriad of potential synaptic partners. How molecular recognition and neural activity cooperate to regulate GABAergic synapse formation and maturation remains poorly understood. As a direct mediator of neural activity, neurotransmission not only reflects the functional status of synaptic contacts but also provides the appropriate spatial and temporal precision in guiding synapse formation. Accumulating evidence indicates that, similar to the role of glutamate at developing excitatory synapses, GABA may coordinate pre- and post-synaptic maturation at inhibitory synapses (Chattopadhyaya et al., 2007; Huang, 2009; Fu and Huang, 2010; Wu et al., 2012), but the underlying mechanisms are yet to be elucidated.

GABA transmission is mediated by both the ionotropic GABA_A_ and metabotropic GABA_B_ receptors (GABA_B_Rs). In addition to mediating slow inhibition, GABA_B_Rs have been implicated in aspects of neural development (Lujan et al., 2005), including axon morphogenesis (Xiang et al., 2002). For example, GABA_B_R signaling results in the inhibition and endocytosis of presynaptic calcium channels (Puckerin et al., 2006; Tombler et al., 2006); and calcium dynamics have been shown to regulate growth cone and filopodia morphogenesis (Henley and Poo, 2004). Presynaptic GABA_B_Rs may further regulate effectors in addition to calcium channels and cAMP levels (Rost et al., 2011). Here we combined live imaging of cortical GABAergic axons with pharmacology and genetic manipulation to examine the role of presynaptic GABA_B_Rs in inhibitory synapse formation. We found that developing GABAergic axons form significant number of small synaptic vesicle clusters, many of them were mobile. Activation of presynaptic GABA_B_Rs promotes the recruitment and stabilization of vesicle pools in presynaptic boutons, likely through the local enhancement of actin polymerization. Importantly, inactivation of GABA_B_Rs in developing interneurons resulted in aberrant pattern of synapse size distribution, reduced synapse density and axon branching, and reduced frequency of miniature inhibitory currents in postsynaptic pyramidal neurons. These findings begin to link GABA release, presynaptic GABA_B_R signaling, actin polymerization, and regulation of synaptic vesicle pools in the context of activity-dependent inhibitory synapses formation. They suggest that GABA_B_Rs act as a local sensor of the strength of GABA release along developing inhibitory axons and promote the stabilization of stronger release sites through increased recruitment of mobile vesicle pools. Such release-dependent validation and maturation of nascent terminals is well suited to sculpt the pattern of synapse formation and distribution along axon branches.

## Materials and Methods

### Mice and constructs

*Gad67^flx/flx^* mice were a gift from Dr. R. Palmiter (University of Washington). *GABA_B1_^flx/flx^* mice were a gift from Dr. B. Bettler (University of Basel). *PV-ires-Cre* mice were a gift from Dr. S. Arber (Friedrich Miescher Institute for Biomedical Research). All WT and transgenic mice are C57BL/6 genetic background. Constructs with ∼10 kb promoter region of *GAD67* gene is generated as described previously (Chattopadhyaya et al., 2004). *P_G67_-EGFP* was further modified so that EGFP was replaced with either Cre, TdTomato, Synaptophysin-GFP (Syn-GFP), or rat GAD67 gene to generate *P_G67_-Cre*, *P_G67_-TdTomato*, *P_G67_-Syn-GFP*, and *P_Gad1_-GAD67*.

Syn-GFP and DsRed are cloned into *lox-STOP-lox (LSL)* construct using *Xho*I and *Not*I sites. *Cofilin(S3A)-GFP* and *cofilin(S3D)-GFP* constructs were modified to be *cofilin(S3A)-mCherry* and *cofilin(S3D)-mCherry*, and subcloned into the LSL construct. The LSL constructs have CMV and chicken β-actin promoters.

### Slice culture and biolistic transfection

As described previously (Stoppini et al., 1991; Chattopadhyaya et al., 2004), postnatal day 3 (P3) to P5 mouse pups of either sex were decapitated, and brains were rapidly removed and immersed in ice-cold artificial low-sodium CSF (ACSF) containing (in mM) 4 KCL, 5 MgCl_2_, 1 CaCl_2_, 26 NaHCO_3_, 10 glucose, and 8% sucrose, saturated with 95% O_2_/5% CO_2_. Coronal brain slices of the occipital cortex, 400 μm thick, were cut with a Chopper (Stoelting, Wood Dale, IL, USA) into ice-cold ACSF. Slices were then placed on transparent Millicell membrane inserts (Millipore, Bedford, MA, USA), usually two slices/insert, in 30 mM Petri dishes containing 1 ml of culture medium (containing DMEM, 20% horse serum, 1 mM glutamine, 13 mM glucose, 1 mM CaCl_2_, 2 mM MgSO_4_, 0.5 μM/ml insulin, 30 mM HEPES, 5 mM NaHCO_3_, and 0.001% ascorbic acid). Finally, they were incubated in a humidified incubator at 34°C with a 5% CO_2_-enriched atmosphere, and the medium was changed three times per week. The age of the culture is reported as equivalent postnatal (EP) day; for example, EP13 for cultures prepared from P4 mice means P4 + 9 days *in vitro*.

Constructs to be transfected were incorporated into “bullets” that are made using 1 μm gold particles coated with 20–30 μg of each DNA construct of interest (except for actin FRET experiments, in which we used 25 μg *LSL-actin-CFP* and 75 μg *LSL-actin-YFP*). These bullets were used to biolistically transfect slices by gene-gun (Bio-Rad, Hercules, CA, USA) at high pressure of helium, and the transfected slices were incubated for 3–4 days under the same conditions as described above, before imaging.

### Two strategies for labeling and manipulating PV basket neurons

In most of the experiments, we used *PV-ires-Cre* mice and *LSL* conditional expression construct; this strategy is highly specific and robust to label and express gene of interest in PV basket cells (Fu and Huang, 2010). To knockout genes in single basket cells (e.g., GAD67 and GABA_B_R) we used slice cultures from *GABA_B1_^flx/flx^* and *GAD67^flx/flx^* mice transfected with *P_G67_* driven Cre and fluorescent proteins constructs.

### Immunostaining

Mice were anesthetized with sodium pentobarbital (50 mg/kg) and perfused with 4% paraformaldehyde (PFA). Brain sections (50 μm in thickness) were prepared with a vibratome and then blocked in 10% NGS (Normal Goat Serum) and 1% Triton X-100 in PBS. Slices were then immunostained with anti-Parvalbumin antibody (mouse 1:1000, Sigma) followed by Alexa594-conjugated anti-mouse IgG (Molecular Probes, 1:400) and mounted in Vectashield mounting medium (Vector). Confocal images (Zeiss LSM510) were taken using a 63× oil objective (Zeiss NA 1.4). Scans from each channel were collected in multiple-track mode and subsequently merged.

### Biocytin loading and staining

AAV-LSL-DsRed virus was injected into the visual cortex of P30 male mice. One week after *in vivo* virus injection, acute cortical slices were prepared as described previously (Lu et al., 2007). PV basket neurons labeled with AAV-LSL-DsRed were identified first by red fluorescent signal and then by its fast-spiking property. Red basket neurons filled with biocytin (0.2%) through the recording pipette were incubated at 4°C overnight with 4% PFA in PBS, pH = 7.4. After the fixation, slices were rinsed in PBS (5 min for three times), and then incubated overnight with Alexa Fluor^®^ 488 conjugated streptavidin (1:1000; molecular probes, invitrogen) with 0.3% Triton X-100 in PBS. Slices were then rinsed in PBS (5 min for three times) and mounted in Vectashield mounting medium (Vector).

### Axon tracing and reconstruction

Confocal images of the basket cell axon arbor were taken using a 63× oil objective (Zeiss, NA 1.4) and a Zeiss LSM510 confocal microscope. Scans from each channel were collected in multiple-track mode and subsequently merged. Z-stacks were acquired with 1 μm steps, exported as TIFF files, and analyzed using Neurolucida software. The axon was traced and the branching and bouton were marked as described in Neurolucida manual. To trace axons, we first identify some characteristic boutons formed by PV neurons, which are stretch of round swelling structure significantly wider than the interconnecting axon shafts. We then follow them back to find the main stem of the axon. We know that each PV neuron only sends out one axon from the cell body, therefore we can follow the axon branches after identifying the main stem of the axon from the cell body.

### General two-photon imaging

Living slice preparations were imaged using a custom-built two-photon laser scanning microscope based on a Fluoview laser scanning microscope (Olympus America Inc., Melville, NY, USA). The light source was a Ti-Sapphire laser (Chameleon Ultra, Coherent) running at a wavelength of 910 nm. The laser power was monitored by custom-built power meter. Fluorescence was detected in whole field detection mode with a photomultiplier tube (Hamamatsu, Bridgewater, NJ, USA). In general, laser power was adjusted so that additional power failed to reveal previously undetected boutons. Optical sections were collected at 0.5 μm spacing. Slices were kept in a transparent chamber with ACSF (containing in mM: 2.5 KCL, 1 MgSO_4_, 2 CaCl_2_, 25 NaHCO_3_, 1.25 NaH_2_PO_4_, 126 NaCl, and 14 glucose) saturated with 95% O_2_/5% CO_2_. Other drugs were used in the perfusing ACSF as: 10 μM CGP46381 (Tocris, Ellisville, MI, USA), 10 μM baclofen (Tocris, Ellisville, MI, USA).

Slices were allowed to sit in the imaging chamber for half hour before imaging experiments. Full three-dimensional (3-D) image stacks were acquired using a 60× 0.9 NA objective lens at 5× digital zoom (Fluoview software; Olympus), ∼70 nm per pixel. Each image plane was resampled three times and spaced 0.5 μm in the *Z*-dimension. For imaging red and green fluorophores, we used ET517/65M and HQ 620/60M-2P filters, together with a 570 DCXRU dichroic mirror. For imaging CFP and YFP, we used HQ 480/40M-2P and HQ 540/40M-2P filters, together with a 510 DCLP dichroic mirror.

### Analysis of puncta dynamics

An area of axon arbor containing 50–100 boutons was selected to image either every 15 min or every 1 min for an hour. Image stacks were limited to no more than 20 μm thick to minimize overlapping of boutons in projected image. Images were analyzed by custom written MatLab programs. Images in the manuscript were displayed using maximum value projection across the *Z*-axis. The Syn-GFP signal stronger than the background level + 2(Standard Deviation of background) was recognized as true signal. Images were thresholded based on the brightest (top 10%) and dimmest (bottom 10%) pixels in the image. After thresholding, puncta smaller than 0.04 μm^2^ or bigger than 9 μm^2^ were excluded for further analysis. The size and average fluorescent intensity of each punctum was then quantified and recorded. To count lost puncta, thresholded puncta were numbered and compared with consecutive time series images manually and re-examined by another person.

Because an entire dynamic episode (ON-OFF cycle) on average lasts more than 13 min, images at every 15 min interval were taken for quantification of “*% lost puncta per hour*.” Because puncta may repetitively appear on certain sites, different sampling frequency will produce different results. With 15 min interval, only five images were taken for quantification (0, 15, 30, 45, and 60 min). By comparing each one of these time points (0, 15, 30, and 45 min) with the next time point, the number and the identity of each *lost puncta* was recorded. By adding up the number of lost puncta in all these four time points, a total number of lost puncta, *N*_lost_, was obtained. The total number of puncta in the first time point (0 min) was determined as *N*_initial_. The final value appeared in the figure, “*% lost puncta per hour*,” was defined as (*N*_lost_/*N*_initial_) × 100. If there was significant shift of images along time, extra care was taken to only analyze the region that was present in all time points.

“*% recur puncta*,” were obtained by analyzing 1 min interval movies. For each puncta which repetitively appeared on the same site (regardless of how many times it reappeared during the movie), it was counted as one “*recur puncta*.” The total number of “recur puncta” was determined as *N*_recur_. It should be noted that each “reoccur puncta” could produce more than one count in the analysis of the “% of lost puncta,” because it may go through several “episodes” of dynamic cycle (a cycle of appearance and the following disappearance). The total number of puncta in the first time point (0 min) was determined as *N*_initial_. The final value appeared in the figure, “*% recur puncta per hour*,” was defined as (*N*_recur_/*N*_initial_) × 100. If there was significant shift of images along time, extra care was taken to only analyze the region that was present in all time points.

For movies with shorter time interval, appropriate size of imaging area was chosen so that the whole *Z*-stack could be scanned in the designated time interval. The 3-D image on each time point was processed by custom MatLab program to generate maximum projection on each time point and then converted to a movie.

To quantify the correlation between the fluorescent intensity of two neighboring puncta, movie files comprising maximum projection at each time point were used. The fluorescence signal of each bouton was quantified using ImageJ for all 60 time points. For any time point, the correlation was calculated using the fluorescent signal values of these two neighboring sites on immediately previous five time points. Therefore, for a movie with 60 time points, we would obtain the correlation value of the later 55 time points.

### Actin FRET imaging and analysis

**Figure A1:**
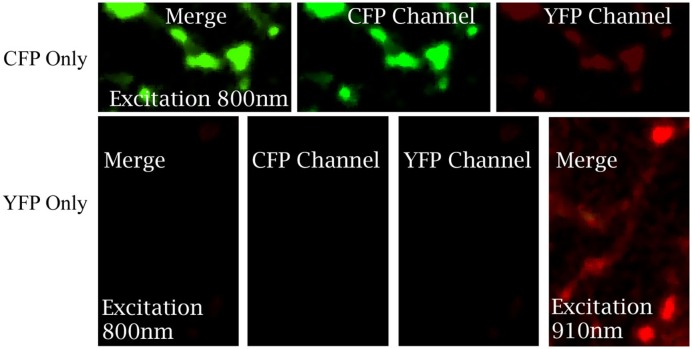
**Imaging of neurons expressing either actin-CFP or actin-YFP alone to estimate the bleed-through between two channels under different excitation wave length**. PV neurons in cortical slice culture were transfected with *LSL-actin-CFP* or *LSL-actin-YFP* at EP14. The labeled neuron was imaged with two-photon microscopy at EP18. Images were taken under different wavelength excitation indicated in the figure.

*PV-ires-Cre* mouse slice cultures were transfected with *LSL-actin-CFP* and *LSL-actin-YFP* (see Biolistic Transfection for detail) at EP15-17 and imaged at EP19-21. Some cultures were also transfected with *LSL-actin-CFP* only or *LSL-actin-YFP* only to determine the bleed-through between two channels and excitation efficiency at different wavelengths, which were later used to determine the YFP/CFP ratio (Figure A1 in Appendix). As described previously (Okamoto and Hayashi, 2006), YFP had no significant emission under 800 nm excitation. Because the slices showed many auto-fluorescent puncta under 800 nm, the selected regions were first imaged at 910 nm to get a clear image of boutons, and then imaged at 800 nm to detect FRET signal. Both the images taken under 800 and 910 nm were processed by custom MatLab programs and the boutons in the 800-nm image were cross-validated by the 910-nm image. All boutons with identifiable signal were included in the data.

The slices were placed in the imaging chamber for 30 min before taking the first image in “control” condition. The CGP was then added into the bathing solution and the second image of “after CGP” was taken 30 min after perfusing in CGP. Slice were then treated, under the presence of CGP, with 10 μM Latrunculin A for 10 min or 10 μM Jasplakinolide for 15 min before taking another image.

### Syn-SEP imaging and stimulation

PV-ires-Cre mouse slice culture was transfected with LSL-syn-SEP and LSL-TdTomato. Whole-cell patch clamp was performed for the syn-SEP/TdTomato expressing basket neuron. Successful patch of transfected basket neuron was confirmed by little back-filling of red fluorophore into the patching pipette. Continuous *X*-*Y* 2-D scanning was performed.

### GABA iontophoresis

The iontophoresis experiment was performed as described previously (Hao et al., 2009). The sharp iontophoretic pipette was filled with 350 mM GABA (pH 3.5, adjusted with HCl) and had a resistance of 150–300 MΩ. The pipette tip was coated with Sylgard-184 (Dow Corning, Midland, MI, USA) to reduce the pipette capacitance. Both the holding current (1.5–2.0 nA) and iontophoretic current (50 nA, with a duration of 1.0 ms) were applied through a Multiclamp 700A amplifier (Molecular Devices) and the pipette capacitance was compensated by a built-in function of the amplifier. The perfusing ACSF contained 1 μM TTX, 20 μM NBQX, 50 μM APV, and 50 μM picrotoxin. All drugs are from Tocris Bioscience. The iontophoretic pipette was also loaded with Alexa 594 (Invitrogen). A small stretch of basket axon with *Z*-axis <3 μm was selected for imaging. The boutons were first imaged under 800 nm to collect initial FRET level, and then another image was collected 2 s after the iontophoresis. Each experiment was repeated five times with 5 min interval, and the final FRET level change for each bouton was the average of the five repeated experiments.

### mIPSC recording

Coronal sections (250 μm) containing primary visual cortex were cut from P40 male mice using a Leica VT1000S vibratome in ice-cold choline dissection media (25 mM NaHCO3, 1.25 mM NaH2PO4, 2.5 mM KCl, 7 mM MgCl2, 25 mM glucose, 0.5 mM CaCl2, 110 mM choline chloride, 11.6 mM ascorbic acid, 3.1 mM pyruvic acid). Slices were incubated in artificial cerebral spinal fluid (ACSF, contains 127 mM NaCl, 25 mM NaHCO3, 1.25 mM NaH2PO4, 2.5 mM KCl, 2 mM CaCl2, 1 mM MgCl2, 25 mM glucose) immediately after cutting, and allowed to recover for at least 1 h at room temperature. All solutions were saturated with 95% O2, 5% CO2, and slices were used within 6 h of preparation.

Whole-cell voltage clamp recordings were performed in ACSF at room temperature from layer II/III pyramidal neurons in primary visual cortex. Recording pipettes were pulled from borosilicate glass capillary tubing with filaments to yield tips of 3–5 MΩ resistance. Neurons were identified by cell body position and visual morphology under infrared DIC optics.

For mIPSC recordings the internal solution contained 147 mM CsCl, 5 mM Na2-phosphocreatine, 10 mM HEPES, 2 mM MgATP, 0.3 mM Na2GTP, and 1 mM EGTA. Osmolarity was adjusted to 300 mOsm with water and pH was adjusted to 7.3 with CsOH. mIPSCs were pharmacologically isolated by bath application of 1 μM TTX (Tocris Bioscience), 50 μM APV (Tocris Bioscience) and 10 μM NBQX disodium salt (Tocris Bioscience). The membrane potential was held at −75 mV and events were filtered at 5 kHz. The sampling rate was 20 kHz. The mini events were detected using MiniAnalysis program with the threshold for detection of an event set at the level three times higher than the root-mean-square noise. Cells with series resistance larger than 20 MΩ were discarded. All electrophysiological recordings were performed and analyzed blind to genotype.

### Curve fitting

For Figures 2E,F and 3E,F, the curving fitting was done by custom written MatLab program using least square curve fitting algorithm and the equation: *y* = *a* × e^(−*x*/*b*)^.

### Statistics

Results are shown as Mean ± SEM, statistical differences between two groups of data were evaluated using Student’s *t*-test, except otherwise mentioned in the text. All experiments were performed with at least three independent replicates. Differences were considered significant for *p* < 0.05.

## Results

### Dynamic exchange of synaptic vesicle pools among developing presynaptic boutons

**Figure 1:**
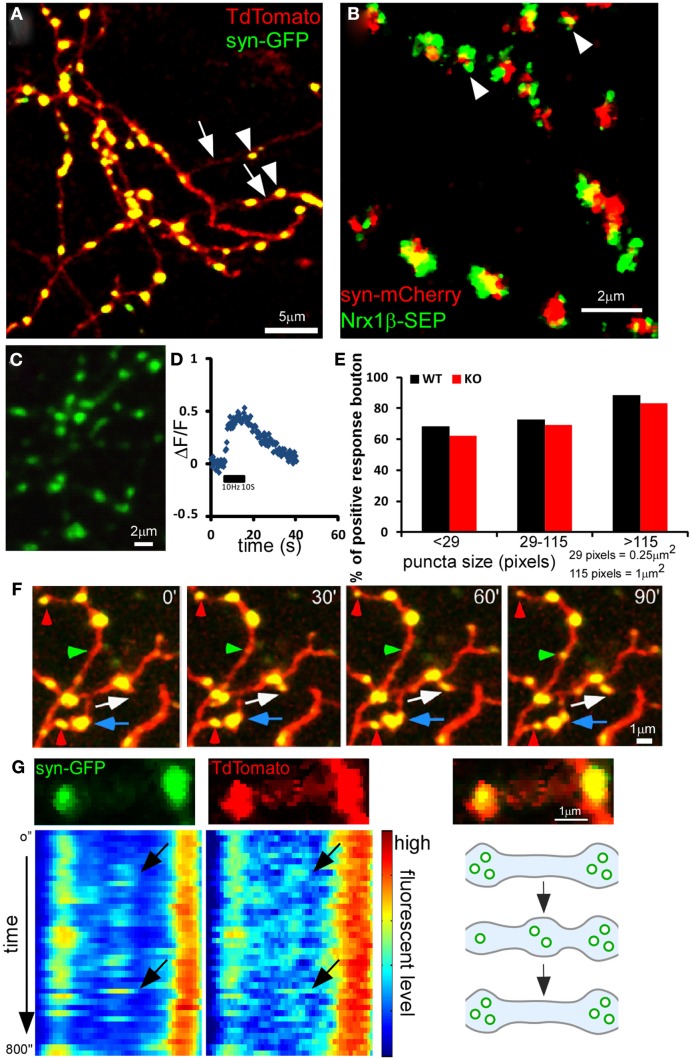
**Dynamics of presynaptic boutons and synaptic vesicle pools in developing GABAergic axons**. Cortical organotypic slice cultures were biolistically transfected at ∼EP14 and two-photon images were taken at ∼EP18 at 910 nm for GFP or 990 nm for mCherry. Representative images are shown. **(A)** Axons of a basket neuron transfected with *P_G67_-TdTomato* and *P_G67_-syn-GFP*. Arrows indicate axon shafts and arrowheads indicate boutons and syn-GFP puncta. **(B)** Axons of a basket neuron transfected with *Lox-STOP-Lox(LSL)-NRX1*β*-SEP* and *LSL-syn-mCherry* in slice cultures from *PV-ires-Cre* mice. Note that almost all syn-mCherry puncta contain NRX1β-SEP (arrowheads). **(C)** Axons of a basket neuron transfected with *LSL-syn-SEP* using slice culture of *PV-ires-Cre* mice. **(D)** Representative fluorescent level changes on a single bouton along a basket cell axon expressing syn-SEP, after a 10s 10 Hz stimulation. **(E)** Syn-SEP was expressed in PV-ires-Cre slice culture from EP15, and the expressing neurons were patch clamped and imaged at EP20. Fluorescent changes on single boutons were recorded during and after 10s of 10 Hz stimulation. Boutons showed significant positive increase of fluorescent was quantified for both WT (182 boutons from seven cells) and *GABA_B1_^flx/flx^::PV-ires-Cre* slice culture (218 boutons from seven cells). Fisher’s-exact test of the proportion of functional boutons in both WT and GABA_B_R KO neurons in different size population showed no significant difference. **(F)** Axon terminals in **(A)** were imaged at 30 min intervals. Red arrowheads indicate boutons that shrink and diminish their syn-GFP signal. Green arrowheads indicate bouton that enlarge and accumulate syn-GFP. The blue arrow indicates newly formed boutons that split from a larger bouton. The white arrow indicates a newly formed bouton on a newly grown branch. **(G)** The dynamics of syn-GFP puncta closely correlated with that of TdTomato. The top row shows a small portion of an axon branch bearing two boutons (TdTomato) containing syn-GFP puncta (left) and the merged view (right). The kymographs show the dynamic changes of syn-GFP and TdTomato signal in the two boutons in a 800-s movie with 20 s interval. Each horizontal line in the kymograph represents the signal from one time point. Note the moment-to-moment correspondence of signal fluctuations in syn-GFP and TdTomato (black arrows). The bottom right panel shows a schematic of the dynamic changes in **(G)**.

We have developed a cortical organotypic culture system which recapitulates many aspects of basket interneuron development and allows single cell labeling and genetic manipulations (Chattopadhyaya et al., 2004). Between EP day 14 (P5 + 9 days *in vitro* and 24, basket interneurons undergo extensive axon and synapse growth toward achieving their characteristic perisomatic innervation pattern (Chattopadhyaya et al., 2004). Depending on specific experimental purposes, two different strategies were used to specifically label and manipulate PV basket neurons in slice cultures. First, a 10-kb promoter region of the *GAD67* gene (*P_G67_*) allows preferential biolistic transfection of basket cells and simultaneous single cell labeling and gene deletion (Chattopadhyaya et al., 2007; Fu and Huang, 2010); second, a Cre-dependent loxp-STOP-loxp cassette allows specific expression in basket cells from slice cultures of *PV-ires-Cre* knockin mice (see Materials and Methods; Fu and Huang, 2010). Here we first used biolistic co-transfection of the *P_G67_-tdTomato* and *P_G67_-synaptophysin(syn)-GFP* constructs to simultaneously label basket cell axon morphology (tdTomato) and synaptic vesicle pools (syn-GFP), respectively (Figure 1A). We have previously shown that the vast majority of RFP-filled axonal boutons (95.3%) contain syn-GFP at EP18, most syn-GFP labeled boutons co-localized with vGAT and were opposite to putative postsynaptic gephyrin clusters and GABA_A_ receptors, and nearly all boutons are synaptic contact sites (Chattopadhyaya et al., 2007; Wu et al., 2012). Here we found that the size of syn-GFP puncta correlated well with the size of the corresponding bouton (Figure 1A). Further, almost all (96.13 ± 1.31%) syn-mCherry labeled boutons contained the key synaptic adhesion molecule neurexin1β (NRX1β), visualized as a NRX1β-SEP (Super-Ecliptic-GFP, pH sensitive GFP) fusion protein (Figure 1B), which localizes to presynaptic boutons through binding to postsynaptic ligands (Fu and Huang, 2010). To further investigate the functionality of these syn-GFP puncta, we expressed SEP-tagged synaptophysin (syn-SEP) and examined the fluorescent change on these puncta upon electrical stimulation (Burrone et al., 2006; Armbruster and Ryan, 2011). The axonal distribution pattern of syn-SEP resembled that of syn-GFP (Figure 1C). With whole-cell patch clamp of the transfected basket cells and 10 Hz stimulation for 10 s, most syn-SEP puncta showed significant increase of fluorescence, indicating functional recycling of synaptic vesicle and releasing of GABA (Figures 1D,E). On average, over 70% of syn-SEP puncta showed significant increase of fluorescent level (Figure 1E). Although we cannot rule out the possibility that certain small syn-GFP puncta could represent axonal transport packets, together the above evidence indicates that in our system, as in other developing circuits (Nakata et al., 1998; Meyer and Smith, 2006; Ruthazer et al., 2006), syn-GFP puncta is a proxy of potential synaptic contact sites. Because the accumulation of synaptic vesicles (SV) on potential synaptic sites is a crucial step during synaptic formation (Waites et al., 2005), studying the dynamics of synaptic vesicle pools, represented by syn-GFP puncta, may reveal the dynamics of synaptic formation and maturation.

At EP18, basket interneurons have already elaborated significant axon arbor with extensive branches that innervate target neurons (Chattopadhyaya et al., 2004). Basket axons continue to extend local branches that innervate new targets and also begin to extend “terminal branches” which eventually form clustered perisomatic synapses around the pyramidal cell soma (Chattopadhyaya et al., 2004). Since vertebrate synapse assembly proceeds in the time course of minutes to hours (Ahmari et al., 2000; Ruthazer et al., 2006; Sabo et al., 2006), we used live cell two-photon imaging to examine the dynamics of basket cell axons in the minute-range time resolution. While the majority of syn-GFP puncta and RFP boutons were stable in the course of 1.5 h, a subset displayed significant dynamic changes: they disappeared, appeared, or split during the same period (Figure 1F). By imaging at higher temporal resolution (every 20 s), we found that the dynamic changes of syn-GFP puncta correlated closely with the corresponding RFP boutons (Figure 1G and Movie S1 in Supplementary material). Kymograph analysis revealed that syn-GFP signals moved as discrete packets and tended to appear and disappear repeatedly at the same sites (Figure 1G; note the repeated occurrence of hot spot on the middle site in the kymograph). To exclude signals close to the resolution of two-photon microscopy, we restricted our analysis to syn-GFP puncta larger than 5 pixels in size (about 0.2 μm in diameter).

**Figure 2:**
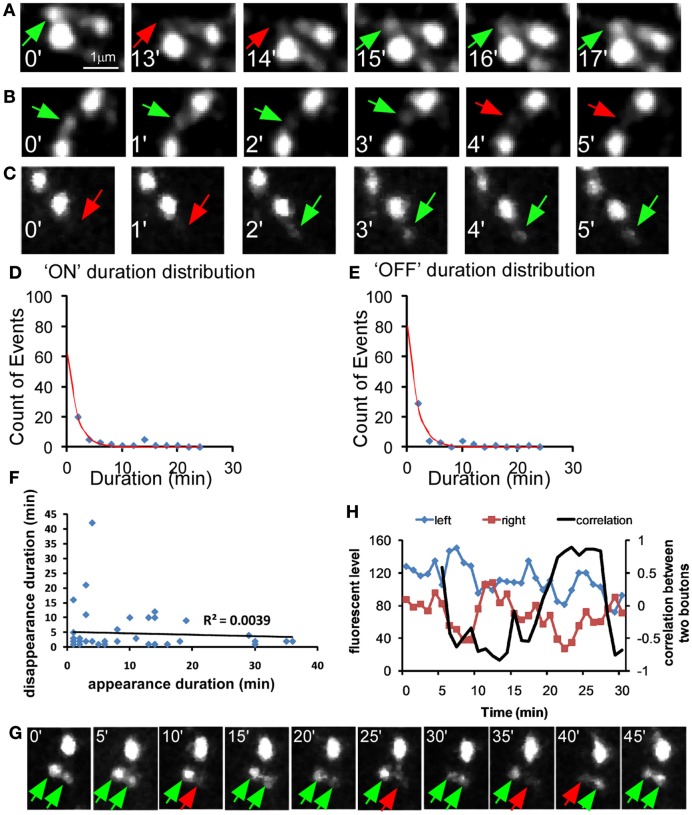
**Dynamic behavior of syn-GFP puncta among neighboring boutons in developing GABAergic axons**. PV basket neurons were transfected with *LSL-syn-GFP* and *LSL-DsRed* in slice cultures from *PV-ires-Cre* mice and their axon terminals were imaged for 1 h at 1 min interval. **(A)** A “recur” puncta which disappear (red arrow) and appear (green arrow) within several minutes. **(B)** A “Disappear” puncta. **(C)** A “New” puncta. **(D–E)** For the recur puncta events in Figure 2D, the distribution of “ON” and “OFF” duration was plotted and fitted with *y* = *a* × exp(−*x*/*b*) function (blue dots, raw data; red line, fitted line). **(F)** For each “recur” puncta, the appearance (ON) and the following disappearance (OFF) duration was plotted for each episode. The linear regression was applied for the data and the *R*^2^ was shown (45 data points from five cells). There was no correlation between the length of ON and OFF duration (*P* = 0.68, ANOVA regression analysis). **(G)** Two neighboring syn-GFP puncta showing reciprocal changes in intensity in tens of minutes. **(H)** Changes in syn-GFP signal at two neighboring sites in **(G)** were plotted with time (blue and red curves). The correlation of changes between the two sites was also plotted (black curve; see Materials and Methods).

Using a 1-min interval imaging protocol (Movie S2 in Supplementary Material), we found that while the large majority (93.06%) of the syn-GFP puncta were stable in a 1-h session, there was a significant portion (5.9%) that repeatedly appeared and disappeared at the same site; we refer to these as “recur puncta” (Figures 1G and 2A). In addition, there were two more rare populations that either disappeared or appeared in the 1-h session (Figures 2B,C; Table 1). These results suggest that developing basket cell axons likely make a large number of transient contacts over the course of several days during which perisomatic synapses form and mature. On average, the “presence (ON) duration” of recur puncta was 8.44 ± 1.48 min and the “absence (OFF) duration” was 4.73 ± 1.08 min (*n* = 45). The distribution of both “ON” and “OFF” duration for all observed events is exponential, suggesting a Poisson distribution process (Figures 2D,E). For each episode of these recur puncta (a full ON-OFF cycle), there was no significant correlation between the length of ON and OFF duration (*P* = 0.68, ANOVA regression analysis; Figure 2F), suggesting that the developing axon repeatedly tested these presynaptic sites, independent of previous attempts, by accumulating SV and probably by transmission. Interestingly, the disappearance of a recur punctum at one site resulted in a significant increase or even appearance of syn-GFP signal in a neighboring site (Figures 1G and 2G,H). Our results suggest that developing GABAergic axons continuously test a large number of potential synaptic sites by recruiting and dispersing synaptic vesicle pools that are probably redistributed among nearby sites. Our current data do not allow us to distinguish whether there was dynamic exchange of syn-GFP puncta among neighboring boutons or whether small syn-GFP packets accumulate or leave independent of their location in relation to nearby boutons. It should also be noted that whether the recur/lost puncta are actually opposite any postsynaptic markers or co-localized with cell adhesion molecules is not clear.

**Table 1:** **Number of observations**.

	WT		KO	
	Number	%	Number	%
Stable	268	93.06	326	88.11
Recur	17	5.903	39	10.54
Disappear	2	0.694	3	0.811
New	1	0.347	2	0.541
Total	288		370	

**Figure 3:**
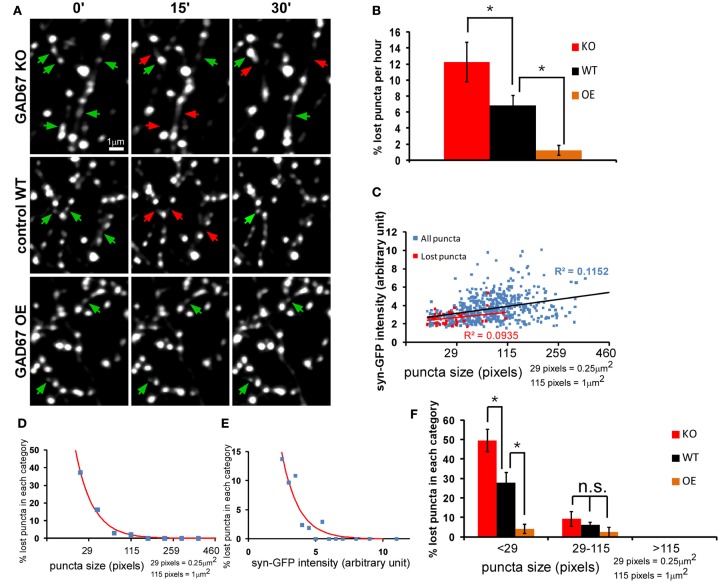
**The strength of GABA transmission regulates the stability of nascent syn-GFP puncta**. Cortical slice cultures of *GAD67^flx/flx^* mice were biolistically transfected at ∼EP15 with *P_G67_-syn-GFP* and *P_G67_-TdTomato*. *GAD67* overexpression (OE) was achieved by including an additional *P_G67_-Gad67* construct. Single basket cell *GAD67* knockout was achieved by including an additional *P_G67_-Cre* construct. **(A)** Sparsely labeled basket neurons were imaged at EP18-20 at 15 min interval for 1 h. Representative images of the first half hour were shown. Red arrows indicate the disappeared puncta, and green arrows indicate appeared and persistent puncta. **(B)** The percentage of lost puncta was analyzed as described in the Section “Materials and Methods.” (KO: 6 cells, 476 puncta; WT: 5 cells, 565 puncta; OE: 4 cells, 406 puncta; one-way ANOVA *P* = 0.006, *post hoc* Dunn’s test, *p < 0.05) **(C)** The syn-GFP puncta size vs. signal intensity for all puncta (blue dots) and for lost puncta (red dots) in WT basket cells (565 puncta for all puncta, 39 lost puncta). Linear regression was applied for both groups and *R*^2^ were displayed. **(D)** The size of all syn-GFP puncta and lost puncta of WT cells was binned. The number of lost puncta in each category was divided by the number of total syn-GFP puncta in that category to derive the percentage of lost puncta in that category. The red line is the fitted curve with function *y* = *a* × exp(−*x*/*b*). **(E)** The intensity of all syn-GFP puncta and lost puncta was binned with arbitrary unit. The number of lost puncta in each category was divided by the number of total puncta in that category to calculate the percentage of lost puncta. The red line is the fitted curve with function *y* = *a* × exp(−*x*/*b*). **(F)** Both the whole syn-GFP puncta population and the lost puncta population were classified according to their size. The percentage of lost puncta in each category was determined as the number of lost puncta in that category divided by the number of total puncta in that category. Significant change was only found in the <29-pixels group (one-way ANOVA *P* = 0.0002, *post hoc* Dunn’s test, *p < 0.05).

Since one episode of recur puncta lasts ∼13 min (average ON-OFF cycle), we conducted most of the imaging experiments using a 15-min interval protocol. By comparing the presence of individual puncta at every 15 min, we quantified the number and percentage of “lost puncta” in an imaging session as a measure of presynaptic vesicle pool stability (see Materials and Methods). We found that lost puncta accounted for 6.84 ± 1.2% of the total initial puncta in a 1-h imaging session (Figures 3A,B, control group). By analyzing the correlation among size, intensity, and stability, we found that the lost puncta clustered into the group with small size and low intensity, and there was weak but significant correlation between puncta intensity and size for the whole puncta population (*P* < 0.0001, ANOVA regression analysis; Figure 3C). Importantly, the percentage of lost puncta increased exponentially with decreasing size, and ∼37.2% of the puncta with size smaller than 29 pixels were lost (Figure 3D). We have to keep in mind that the resolution of two-photon microscope is close to 0.5 μm. Therefore, we could not distinguish some smaller puncta that are very close. This might lead to over-estimation of the stability of these smaller puncta. This percentage also increased exponentially with decreasing intensity, as 13.7% of the puncta with intensities less than 2.5 arbitrary units were lost (Figure 3D). Puncta with average intensity (3.64 arbitrary units) corresponded to a 6.1% loss based on the fitted curve (Figure 3E). On the other hand, the average puncta size was 79 pixels, which corresponded to 4.5% loss on the fitted curve. Therefore, a size increase from 29 to 79 pixels reduced the percent of puncta loss from 37.2 to 4.5%, suggesting that the increase of synaptic vesicle pool is a crucial factor that augmented vesicle pool stability.

### GABA signaling through presynaptic GABA_B_R regulates bouton stability

We first examined the role of GABA transmission in regulating presynaptic syn-GFP puncta dynamics and stability by genetic manipulation of GABA levels in, and thus GABA release from, basket interneurons. We have shown that genetic deletion or knockdown of *GAD67*, the rate-limiting enzyme for GABA synthesis, resulted in cell autonomous deficits in perisomatic synapse formation (Chattopadhyaya et al., 2007). Here we show that *GAD67* gene deletion in single basket cells, achieved by biolistic co-transfection of *P_G67_-Cre* and *P_G67_-syn-GFP* using slice cultures from *Gad67^flx/flx^* mice, resulted in decreased syn-GFP puncta stability. Conversely, *GAD67* overexpression (OE) in basket cells using the *P_G67_-Gad67* construct increased their puncta stability (Figures 3A,B). These manipulations mainly influenced the stability of small puncta (Figure 3F), suggesting that GABA transmission is more effective in stabilizing synaptic vesicle pool in small boutons that may represent nascent contacts, likely in a cell autonomous manner. Larger boutons may have recruited other mechanisms (e.g., multiple classes of synaptic adhesion molecules) for their stabilization and therefore became less dependent on GABA transmission.

**Figure 4:**
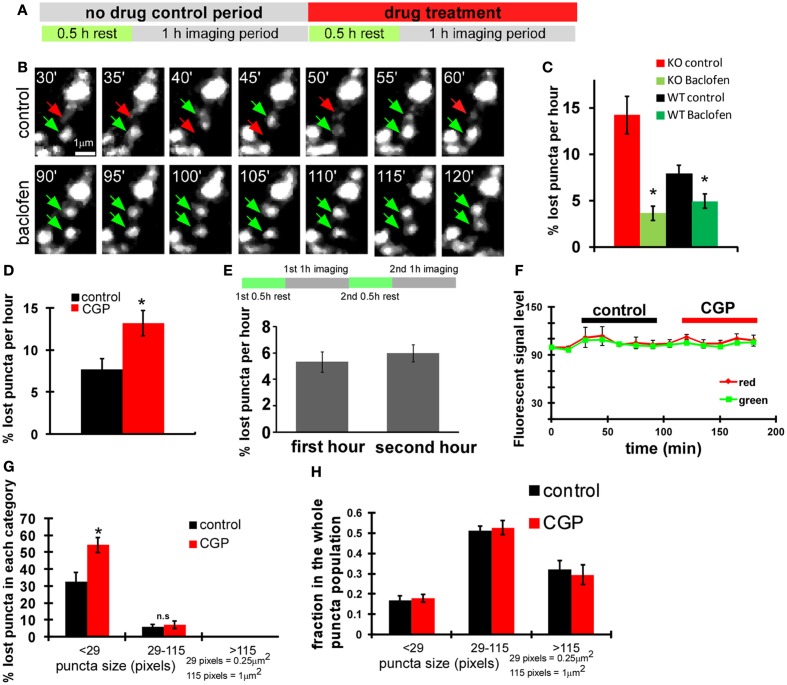
**GABA_B_R in basket cells regulates the stability of nascent syn-GFP puncta**. **(A)** Scheme of imaging experiments with drug treatment. **(B)** Acute baclofen (10 μM) treatment rescued unstable syn-GFP puncta in *GAD67^−/−^* cells transfect with the *P_G67_-Cre* construct using slice cultures from *GAD67^flx/flx^* mice. Representative images at the indicated time points of the same set of puncta were shown. Red arrows and green arrows indicate the disappeared puncta and the present puncta, respectively. **(C)** The percentage of lost puncta was quantified for both WT and *GAD67^−/−^* KO basket neurons under control condition and baclofen treatment (*n* = 7 cells, *t*-test *P* = 0.002 for KO, *P* = 0.026 for WT, comparing with corresponding control group). **(D)** Using slice cultures from *PV-ires-Cre* mice, PV basket neurons labeled by *LSL-syn-GFP* and *LSL-DsRed* were imaged first under control condition and then under 10 μM CGP treatment. The percentage of lost puncta was quantified (*n* = 6 cells, *t*-test *P* = 0.002). **(E)** Bouton dynamics did not change significantly during the second 1 h imaging session under control condition. Cortical PV neurons were labeled with *LSL-syn-GFP* and *LSL-DsRed* using slice cultures from *PV-ires-Cre* mice. The two imaging sessions were conducted as depicted in the diagram, and the lost syn-GFP puncta were quantified (*n* = 3 cells). **(F)** CGP treatment did not significantly quench fluorescence protein signal. For neurons analyzed in **(D,E)**, syn-GFP and RFP intensity on 10 randomly picked boutons were followed for all time points. The change of fluorescence levels on the same bouton was analyzed by normalizing with the initial fluorescence signal, which was normalized as 100. **(G)** For neurons imaged in **(D)**, the percentage of lost puncta was plotted according to their size described in Figure 3F (*n* = 6 cells, *t*-test *P* = 0.001). **(H)** Acute CGP treatment did not change the puncta size distribution. For neurons analyzed in Figure 4D, puncta were classified according to size. The fraction of each size category over the whole puncta population was calculated and plotted. The analysis was done for the 30- and 180-min time points, according to the *X* axis in **(F)** (*n* = 3 cells).

**Figure 5:**
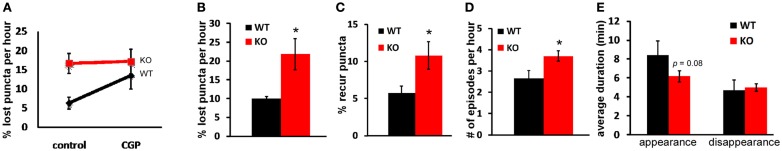
**Presynaptic GABA_B_R in basket cells regulates the stability of nascent syn-GFP puncta**. **(A)** Slice cultures of *GABA_B1_^flx/flx^* mice were biolistically transfected at ∼EP15 with *P_GAD67_-syn-GFP* and *P_GAD67_-TdTomato*. Single basket cell *GABA_B1_* knockout was achieved by including an additional *P_GAD67_-Cre* construct. Labeled neurons were imaged first under control condition and then under CGP treatment. The percentage of lost puncta was quantified (*n* = 4). **(B)** Slice cultures from *GABA_B1_^flx/flx^::PV-ires-Cre* mice in which *GABA_B1_* was deleted in all PV neurons were used. PV neurons were labeled by co-transfection of *LSL-syn-GFP and LSL-DsRed* constructs and their axons were imaged under control condition. The percentage of lost puncta was quantified (*n* = 5 cells for both, *t*-test *P* = 0.03). **(C)** For neurons imaged in **(B)**, the percentage of recur puncta (see Materials and Methods) was quantified (*n* = 5 cells, *t*-test *P* = 0.04). **(D)** For neurons imaged in **(B)**, the number of episodes for each recur puncta was analyzed (*n* = 5 cells, *t*-test *P* = 0.03). **(E)** For all the observed episodes, the duration of appearance and disappearance were quantified (*n* = 129 puncta for KO; *n* = 45 for WT).

To examine whether GABA_B_Rs contribute to the GABA regulation of presynaptic stability in basket cell axons, we first used a pharmacological approach. We found that the acute treatment of GABA_B_R agonist baclofen rescued unstable syn-GFP puncta in both WT and *GAD67^−/−^* cells (*P* = 0.002 for KO, *P* = 0.026 for WT, baclofen treated group vs. corresponding control group; Figures 4A–C). Conversely, the GABA_B_R antagonist CGP46381 (CGP) significantly decreased puncta stability in wild type basket cells (*P* = 0.002; Figure 4D). This decrease was not due to deteriorating physiological condition and phototoxicity after longer imaging period (Figure 4E) or potential quenching of fluorescence by CGP (Figure 4F). The fold change of percentage of lost puncta in the <29-pixels group was significantly different from that of the 29- to 115-pixels group (2.03 ± 0.4 vs. 1.23 ± 0.2, *P* = 0.038); therefore CGP selectively decreased the stability of small puncta (size <29 pixels; Figure 4G, black bars) without changing the relative distribution of bouton size (Figure 4H). To investigate the specific role of presynaptic GABA_B_R in basket cells, we deleted *GABA_B1_* gene in single basket cells by biolistic transfection of *P_G67_-Cre* and *P_G67_-syn-GFP* constructs in slice cultures from *GABA_B1_^flx/flx^* mice and imaged syn-GFP puncta dynamics. We found that the syn-GFP puncta in *GABA_B1_^−/−^* basket cells were much more unstable than those in control (WT) cells, and were no longer sensitive to the treatment of CGP (Figure 5A). These results, together with the cell autonomous effects of *GAD67* manipulations (Figure 3), strongly indicate that GABA_B_Rs along basket cell axon regulate the stability of synaptic vesicle pools. To rule out the possibility that the reduced stability of presynaptic vesicle pools in single *GABA_B1_^−/−^* axons may have resulted from a disadvantage in their competition with nearby *GABA_B1_*^+*/*+^ basket axons, we deleted *GABA_B1_* in all PV basket neurons using slice cultures from *GABA_B1_^flx/flx^: PV-ires-Cre* mice. Syn-GFP puncta in these *GABA_B1_^−/−^* PV basket cells were also less stable than those in *GABA_B1_*^+*/*+^ PV basket cells in slice cultures from control littermates (Figure 5B). We further examined whether *GABA_B1_^−/−^* axons contained more non-functioning syn-GFP puncta, assayed as the proportion of syn-SEP puncta showing significant positive change after a train of stimulation. We found no significant difference in the proportion of functional syn-SEP puncta between WT and *GABA_B1_^−/−^* PV neurons in all size populations (Figure 1E), indicating that the lack of GABA_B_R, rather than impaired transmission, accounts for the decreased vesicle pool stability in *GABA_B1_^−/−^* neurons.

The increased loss of syn-GFP puncta in *GABA_B1_^−/−^* axons could have resulted from an increase in the proportion of presynaptic sites which contain unstable puncta and/or an increased turnover rate of recur puncta at the same presynaptic site. When imaging at a 1-min interval, we found that recurring puncta remained as the major component of dynamic events in *GABA_B1_^−/−^* cells compared with those in control cells (Table 1), but the percentage of the recur population was much higher in *GABA_B1_^−/−^* (10.5%) than in WT (5.9%) neurons (Figure 5C, Table 1). Quantification of the dynamics of recur puncta at the same presynaptic site further revealed that there was also a significant increase in the number of episodes during the 1-h imaging period in *GABA_B1_^−/−^* axons (Figure 5D); this was due to a decrease in the ON duration of recur puncta in each episode (8.4 min in WT cells vs. 6.2 min in *GABA_B1_^−/−^* cells; Figure 5E). These two changes, the increased proportion of presynaptic sites containing unstable puncta and increased turnover rate of recur puncta at the same presynaptic site, appear to account for the increased loss of syn-GFP puncta in *GABA_B1_^−/−^* axons. Indeed, by multiplying the fold increase in “proportion of recur puncta” (GABA_B_R*^−/−^*/WT = 1.78, Figure 5C) and the fold increase in “number of episodes per hour” (GABA_B_R*^−/−^*/WT = 1.4, Figure 5D), the result (2.49) is close to the “fold increase in proportion of lost puncta” that we experimentally observed (GABA_B_R*^−/−^*/WT = 2.24, Figure 5B). Altogether, these results suggest that cell autonomous GABA signaling stabilizes synaptic vesicle pools in small boutons through presynaptic GABA_B_Rs.

### Presynaptic GABA_B_R locally regulates actin polymerization in GABAergic boutons

**Figure 6:**
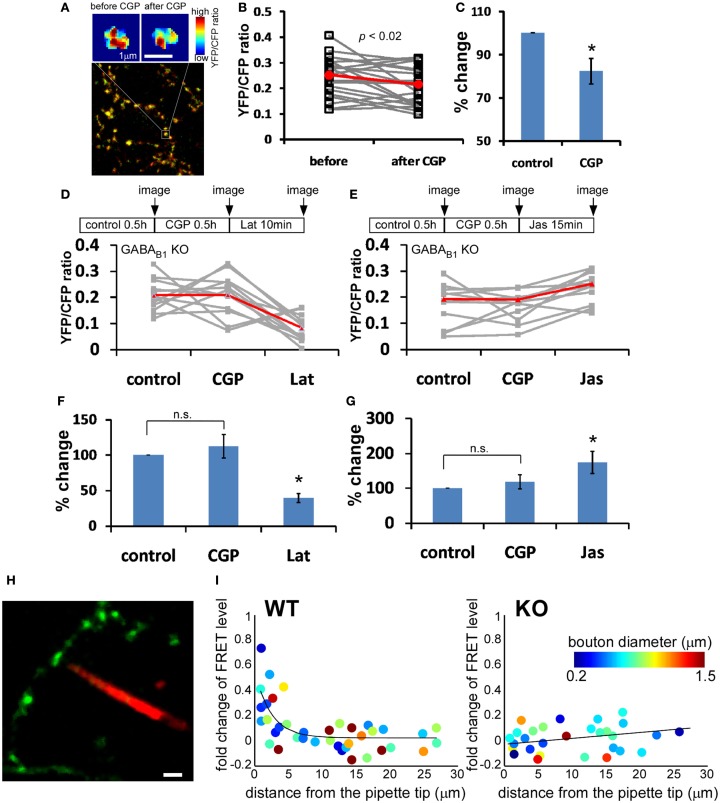
**Presynaptic GABA_B_R locally modulates actin polymerization in GABAergic boutons**. Slice cultures from *PV-ires-Cre* mice were transfected at ∼EP15 with *LSL-actin-CFP* and *LSL-actin-YFP* to express this FRET pairs in PV neurons. Labeled neurons were imaged at EP18–20. **(A)** Representative heat-maps of FRET level in the same bouton under control condition and after 30 min treatment of 10 μM CGP46381 (CGP). Warmer color indicates higher FRET level. **(B)** The YFP/CFP ratio of 24 randomly chosen boutons (gray lines) from three different cells was quantified before and after 30 min CGP treatment. Red line is the average across all boutons. CGP treatment reduced YFP/CFP ratio. **(C)** When normalized with YFP/CFP ratio under control condition, CGP reduced YFP/CFP ratio by 17.6 ± 5.8% (paired *t*-test *P* = 0.004; *n* = 24 from four cells). **(D–G)** *GABA_B_^−/−^* PV neurons in *GABA_B1_^flx/flx^::PV-ires-Cre* slice cultures were labeled by *LSL-actin-YFP and LSL-actin-CFP*. The change in YFP/CFP ratio of each bouton was followed under different drug treatments as indicated (gray lines). Red line is the average across all boutons. Latrunculin A (10 μM, 10 min) or Jasplakinolide (10 μM, 15 min) was added under the presence of CGP. Percent changes in YFP/CFP ratio were normalized to control condition (*n* = 12 from three cells; one-way repeated measures ANOVA, Bonferroni *post hoc* test, *P* < 0.005 for Lat vs. CGP, *P* < 0.01 for Jas vs. CGP). **(H)** Representative image of two-photon imaging-guided placement of iontophoresis pipette tip to close vicinity of a single bouton. **(I)** The change of FRET level after focal iontophoresis of GABA, plotted against the distance between individual bouton and pipette tip (*n* = 6 cells for both WT and KO). Solid lines are fitted curve with *y* = *a* × exp(−*x*/*b*).

GABA_B_R is coupled to G_i/o_ (Bettler et al., 2004), which has been shown to regulate actin dynamics (Welch and Mullins, 2002), and regulation of actin polymerization contributes to activity-dependent maturation of glutamatergic presynaptic terminals (Shen et al., 2006) and the exchange of synaptic vesicle pools among nearby boutons (Darcy et al., 2006). We therefore examined actin polymerization levels in GABAergic boutons by measuring the FRET (Förster resonance energy transfer) levels between actin-CFP and actin-YFP (Okamoto and Hayashi, 2006) by co-transfection of *LSL-actin-CFP* and *LSL-actin-YFP* in slice cultures from *PV-ires-Cre* mice (Figure 6A). Although different boutons in different cells displayed variable basal YFP/CFP ratios, paired comparison before and after CGP treatment showed significant decrease of YFP/CFP ratio (17.7% decrease, *P* = 0.004; Figures 6B,C). This effect was abolished in *GABA_B1_^−/−^* PV neurons in slice cultures from *GABA_B1_^flx/flx^::PV-ires-Cre* mice, indicating that CGP affected YFP/CFP ratios through presynaptic GABA_B_Rs (Figures 6D,E). As a control experiment for the functionality of actin-CFP and actin-YFP pair in these *GABA_B1_^−/−^* PV neurons, we used latrunculin A (Lat) or jasplakinolide (Jas), which directly depolymerizes and polymerizes actin, respectively. YFP/CFP ratios significantly decreased with Lat treatment and increased with Jas treatment under the presence of CGP in *GABA_B1_^−/−^* PV neurons (Figures 6F,G).

To further demonstrate that presynaptic GABA_B_Rs act locally along developing axon terminals, we performed focal GABA iontophoresis and examined actin polymerization levels on individual boutons. Guided by two-photon imaging, we placed the pipette tip loaded with alexa 564 very close to single boutons (Figure 6H). In WT neurons, a 1-ms pulse of GABA produced significant increase of FRET level in boutons closest to the pipette tip. The effect of GABA iontophoresis on FRET levels decreased exponentially in boutons more distant from the pipette tip, and there is almost no effect for boutons 5 μm away from the pipette tip. The effect of iontophoresis was independent of bouton size. In contrast, no FRET change was observed in *GABA_B1_^−/−^* PV neurons, regardless of the distance between bouton and pipette tip (Figure 6I). Together these results indicate that presynaptic GABA_B_R signaling locally promotes actin polymerization in developing GABAergic boutons. The diminishing effect on actin polymerization in boutons with increasing distance to pipette tip further suggested that the amount of GABA release and thus the level of presynaptic GABA_B_R activation quantitatively regulates actin polymerization level.

### Actin polymerization regulates GABAergic vesicle pool stability

**Figure 7:**
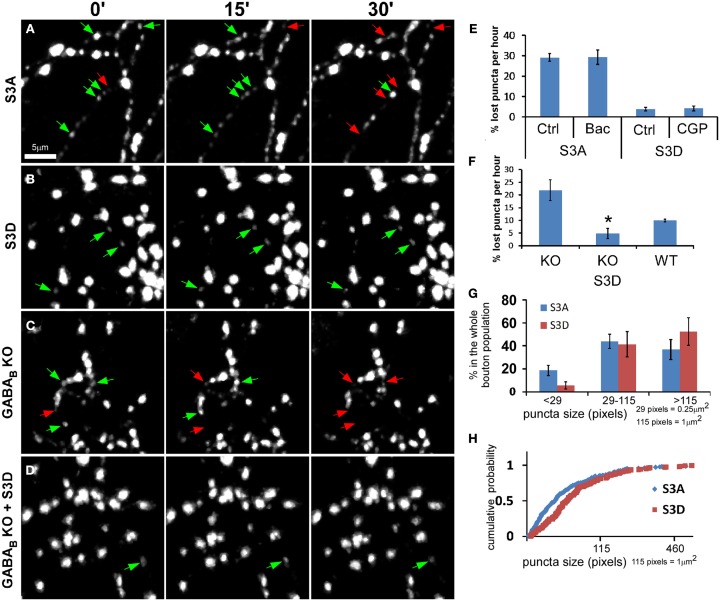
**Actin polymerization levels influence syn-GFP puncta stability in GABA axons**. **(A–B)** PV neurons in *PV-ires-Cre* slice cultures were co-transfected with *LSL-syn-GFP* and *LSL-cofilin(S3A)-mCherry* **(A)** or *LSL-cofilin(S3D)-mCherry* **(B)** between EP15–17. Labeled neurons were imaged for 1 h at 1 min interval between EP20–22. Red arrows and green arrows indicate disappeared puncta and present puncta, respectively. **(C-D)** *GABA_B1_^−/−^* PV neurons in *GABA_B1_^flx/flx^::PV-ires-Cre* cultures were transfected with *LSL-syn-GFP*, together with either *LSL-mCherry* **(C)** or *LSL-cofilin(S3D)-mCherry* **(D)**, and were imaged for 1 h at 1 min interval between P19–21. **(E)** Labeled neurons in **(A)** and **(B)** were imaged first under control (Ctrl) and then under indicated drug treatment. Lost puncta in both control and indicated drug treatments were quantified (*n* = 4 cells for each group). **(F)** Lost puncta in **(C)** and **(D)**, as well as that of WT PV neurons from slice cultures from sibling *GABA_B1_^+/+^::PV-ires-Cre* mice were quantified. The value of KO and WT were re-plotting of Figure 5B (*n* = 5 cells for each group; one-way ANOVA, *post hoc* Dunn’s test, *P* = 0.01 for KO vs. KO S3D). **(G–H)** Actin depolymerization resulted in increased population of small syn-GFP puncta. Cortical PV neurons were transfected with *LSL-syn-GFP*, as well as either *LSL-cofilin(S3A)-mCherry* or *LSL-cofilin(S3D)-mCherry* using slice cultures from *PV-ires-Cre* mice at EP15–17. Bouton size was analyzed at EP20. **(G)** The distribution of different size population of syn-GFP puncta of S3A and S3D transfected neurons (*n* = 5). **(H)** Cumulative distribution of all puncta of S3A and S3D transfected neurons, showing significant divergence for puncta smaller than 1 μm (*K*-*S* test, *P* < 0.0001).

We examined the role of actins in regulating synaptic vesicle dynamics in GABAergic boutons by manipulating actin polymerization using cofilin mutants. Cofilin binds to actin filaments to sever and depolymerize F-actin but is inactivated after phosphorylation (Arber et al., 1998; Yang et al., 1998). S3A-cofilin is a “non-phosphorylatable” mutant and is constitutively active in depolymerizing actin filaments, whereas S3D-cofilin is “phospho-mimic” thus favors actin polymerization and stabilization (Shi and Ethell, 2006). OE of cofilin(S3A) in PV neurons reduced the stability of syn-GFP puncta, leading to increased puncta lost during the 1-h imaging period (Figure 7A and Movie S3 in Supplementary Material); these puncta also showed significantly increased motility/translocation and morphological changes, including frequent splitting of puncta into smaller mobile packets. Unlike the unstable puncta in *GAD67^−/−^* cells that could be rescued by baclofen (Figure 4B), unstable puncta in cofilin(S3A)-expressing cells were not rescued by baclofen (Figure 7E). In cofilin(S3D)-expressing cells, on the other hand, syn-GFP puncta were more stable (Figure 7B and Movie S4 in Supplementary Material), and blocking GABA_B_R with CGP did not decrease their stability (Figure 7E). Importantly, cofilin(S3D) fully rescued the unstable puncta in *GABA_B1_^−/−^* cells (Figures 7C,D,F; Movie S5 and S6 in Supplementary Material). Furthermore, cofilin(S3A)-expressing cells showed significantly higher proportion of smaller syn-GFP puncta compared with cofilin(S3D)-expressed cells (18.7 vs. 5.8% for puncta <29 pixels in size; Figures 7G,H). These data suggested that actin polymerization status influences presynaptic maturation by modulating the accumulation and dispersion of synaptic vesicle pools in small boutons. Together, these results demonstrate that presynaptic GABA_B_R regulates the stability and motility of synaptic vesicle pools in GABAergic terminals through modulating actin polymerization.

### GABA_B_R deficiency in PV neurons results in aberrant density and size distribution of presynaptic terminals

**Figure 8:**
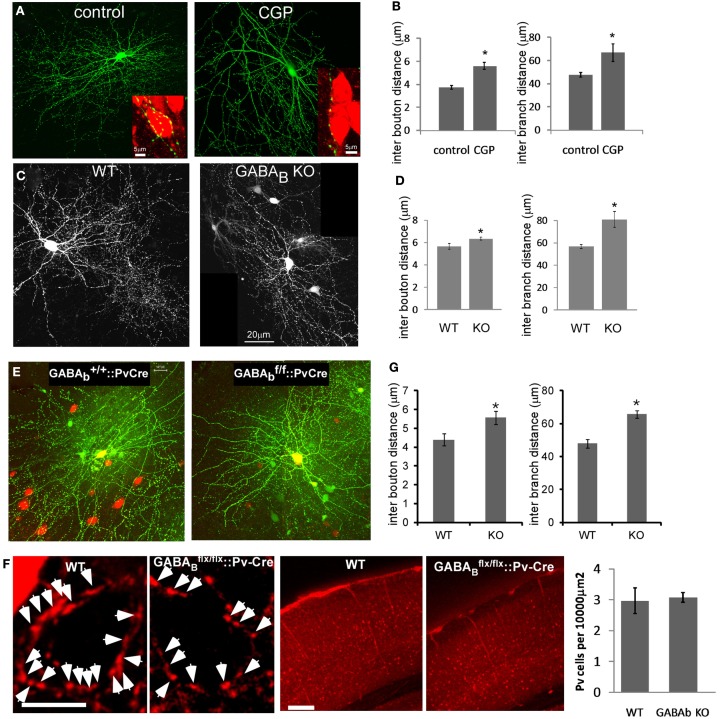
**Blocking GABA_B_R signaling in PV neurons resulted in lower bouton density and less axon branching both *in vitro* and *in vivo***. **(A)** PV neurons from *PV-ires-Cre* slice cultures were labeled by *LSL-YFP*. Slices were treated with CGP from EP14 and fixed at EP19. Inserts show NeuN immunostaining of pyramidal neuron soma (red). **(B)** The axon morphology of PV neurons in **(A)** were reconstructed and analyzed (*n* = 3 cells for each group). **(C)** To delete GABA_B_R in single basket neurons, slice cultures from *GABA_B1_^flx/flx^* mice were transfected with *P_G67_-Cre and P_G67_-GFP* at EP15, and control basket cells were transfected with *P_G67_-GFP*. Slices were fixed at EP19. **(D)** The axon morphology of neurons in **(C)** were reconstructed and analyzed (*n* = 3 cells for each group). **(E)** AAV-LSL-DsRed virus was injected into the visual cortex of *GABA_B1_^+/+^::PV-ires-Cre* or *GABA_B1_^flx/flx^::PV-ires-Cre* mice at P30 to label PV basket neurons. Acute visual cortical slices were prepared at P40 and RFP labeled PV neurons were patch clamped and filled with biocytin. Biocytin loaded PV neurons were fixed and immunostained for biocytin. The stained neurons were imaged by confocal microscopy and the axon morphology was reconstructed using Neurolucida. **(F)** Mice of indicated genotypes were sacrificed and perfused at P40. Coronal sections were immunostained for PV in red. Layer 2/3 of visual cortex was imaged. Arrows indicate the perisomatic PV cell axon terminals. The middle panel shows the lower magnification view of visual cortex stained for PV in red. The number of PV positive neurons was quantified and showed no difference between WT and GABA_B_R KO samples (*n* = 15 regions from three animals). **(G)** The bouton density and axon branching of neurons in **(E)** were fully reconstructed and analyzed (nine WT neurons, eight KO neurons; *P < 0.03).

To link the acute effects of GABA_B_R on the stability of presynaptic terminals to the development of PV cell synapse density and axon arbor, we examined the effect of chronic GABA_B_R blockade by treating slice cultures with CGP from EP14 to EP19. While there was no overt changes of general axon arbor morphology, the linear density of boutons and axon branches decreased significantly (Figures 8A,B). Single basket cell GABA_B_R knockout showed similar phenotypes, suggesting a cell autonomous effect (Figures 8C,D).

**Figure 9:**
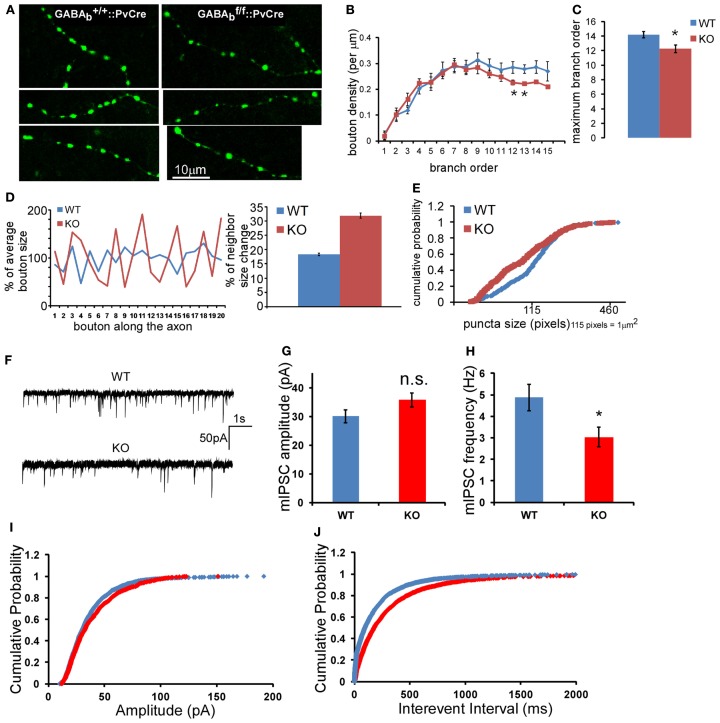
**GABA_B_R deficiency in PV neurons results in aberrant density and size distribution of presynaptic terminals *in vivo***. **(A)** Morphology of axon branches and presynaptic boutons of control (left panels) and *GABA_B1_^−/−^* (right panels) PV neurons in layer 2/3 of visual cortex were labeled by biocytin at P40; mouse genotypes are as indicated. **(B)** The bouton density (# of bouton per μm) on different axon branch order was analyzed for all reconstructed neurons (*P < 0.05, comparing with WT neuron). **(C)** The average maximum axon branch order for all reconstructed neurons (*P = 0.01, *n* = 9 for WT, *n* = 8 for KO). **(D)** The size change of consecutive boutons along one axon branch was plotted for WT and *GABA_B1_^−/−^* KO PV cells. The average bouton size of each branch was normalized as 100%. The percentage of neighbor size change was calculated as described in the Section “Materials and Methods” and was plotted. Data were from 11 axons segments of three cells of both WT and KO neurons, which contained 268 boutons in WT axons and 246 boutons in KO axons (Student *t*-test, *P* < 0.0001). **(E)** Boutons of terminal axon branches of both WT and KO neurons were quantified, and the cumulative probability of bouton size distribution was plotted (332 boutons for WT, 328 boutons for KO; *K-S* test, *P* < 0.0001). **(F)** Sample traces of mIPSC recorded from layer 2/3 pyramidal neurons WT and GABA_B_R KO animals. **(G)** Average mIPSC amplitude of all recorded neurons. **(H)** Average mIPSC frequency of all recorded neurons (*n* = 15 for each group, *P* = 0.02, five WT mice and five KO mice). **(I)** The cumulative distribution of mIPSC amplitude. **(J)** The cumulative distribution of mIPSC inter-event interval (*K-S* test, *P* < 0.001).

To extend these studies *in vivo*, we labeled single basket interneurons in acute brain slices from primary visual cortex in mice in which GABA_B_Rs were deleted in PV interneurons, using biocytin (Figure 8E). In these *GABA_B1_^flx/flx^::PV-ires-Cre* mice, GABA_B_Rs in PV cells were likely inactivated during the second postnatal week, given the onset of PV expression in cortex after the first postnatal week (de Lecea et al., 1995), and should not affect earlier aspects of basket cell development. Indeed, the density and distribution of PV neuron cell bodies in visual cortex of these mice were not altered at ∼P40 (Figure 8F). Reconstruction and quantification of single PV neuron axons revealed that GABA_B_R deficiency resulted in less axon branching, lower bouton density (Figure 8G), and aberrant distribution of presynaptic boutons (Figure 9A). Bouton density on higher order branches or terminal branches were more significantly decreased (Figure 9B), and the maximum branch order of GABA_B_R KO neurons were significantly less than that of the WT ones (14.2 vs. 12.2, *P* = 0.01; Figure 9C). In control (WT) PV neurons, neighboring boutons showed mild and gradual fluctuations in size along axon branches (Figure 9D, WT group). In *GABA_B1_^−/−^* neurons, however, neighboring boutons showed abrupt changes in size (Figure 9D, KO group), and there was a much higher proportion of very small boutons, lower proportion of middle size boutons, and largely unchanged proportion of large boutons compared to those in control cells (*K*-*S* test, *P* < 0.0001; Figure 9E). We further examined the physiological impact of GABA_B_R KO *in vivo* by recording mIPSCs in layer II/III pyramidal neurons in visual cortex. We found that, while the average amplitude of mIPSCs was not significantly changed, the average frequency of mIPSCs was significantly reduced (4.87 ± 0.61 vs. 3.04 ± 0.45 Hz, *P* = 0.02; Figures 9F–J). The more significant decrease of mIPSC frequency than that of bouton density might reflect both the decreased bouton density and higher proportion of small, non-releasing boutons. Together, these results demonstrated that blockade of presynaptic GABA_B_Rs in developing PV neurons *in vivo* resulted in decreased bouton density and axon branching, as well as aberrant distribution and patterning of presynaptic terminals along axons.

## Discussion

A developing cortical basket interneuron axon explores a complex cellular milieu yet eventually achieves characteristic perisomatic innervation, forming clustered synapses around the soma and proximal dendrites of many pyramidal neurons (Tamas et al., 1997; Chattopadhyaya et al., 2004). Our study reveals that developing GABAergic axons form numerous transient boutons and a very small subset of these was stabilized. It is likely that transient contacts reflects a general strategy by which GABAergic axons explore synaptic partners from a myriad of potential targets through cell surface cues as well as neurotransmission. Previous studies suggest that GABA regulates the development of inhibitory synapses (Huang, 2009; Fu and Huang, 2010; Wu et al., 2012). Here we provide evidence that presynaptic GABA_B_Rs act as a local sensor of GABA release along inhibitory axons and coordinate the maturation of presynaptic boutons through the recruitment and redistribution of SV. It has been well-established that GABA_B_R at glutamatergic synapses modulate glutamate releasing by sensing ambient GABA levels of the local network. Our current results reveal an alternative scenario in which GABA_B_Rs at GABAergic terminal senses the level of local GABA transmission and modulate the maturation of GABAergic synapses. Such release-dependent stabilization of nascent synaptic contacts is well suited to sculpt the pattern of inhibitory synapse formation along axon branches and postsynaptic targets.

### Transient contacts and mobile synaptic vesicles along developing GABAergic axons

Synapse formation is a major component of axon arbor growth (Meyer and Smith, 2006; Ruthazer et al., 2006). A developing axon engaged in active synapse formation at multiple growing branches would require continuous supply of synaptic proteins such as cell adhesion molecules and vesicle release machinery. Studies in dissociated glutamatergic neurons have demonstrated that, after their synthesis in the cytoplasm, presynaptic components (e.g., synapse vesicles, active zone protein and matrix proteins) are often pre-assembled into multi-protein complexes, such as piccolo transport vesicles (PTVs) and SV protein transport vesicles (SVTs), which are transported over long distance to remote axonal locations (Ahmari et al., 2000; Krueger et al., 2003; Darcy et al., 2006; Sabo et al., 2006; Tsuriel et al., 2006, 2009). SVTs are capable of glutamate release and can be readily incorporated into nascent synapses (Sabo et al., 2006). In addition, “orphan” synaptic vesicle release sites are formed from existing synaptic sites and are mobile in both anterograde and retrograde directions (Krueger et al., 2003). Axonal contact to a potential target could readily recruit these highly mobile release sites and allow a rapid exploration and validation of nascent contact for synaptogenesis (Krueger et al., 2003). In addition, live imaging studies show that presynaptic proteins and vesicles are continuously lost from and reincorporated into nearby synaptic terminals at time-scales of minutes to hours, and the dynamics of synaptic matrix molecules are dominated by such local protein exchange and redistribution (Darcy et al., 2006; Sabo et al., 2006; Tsuriel et al., 2006; Staras et al., 2010). However, the regulatory mechanisms that control the recruitment and dispersion of these mobile vesicles at presynaptic terminals are unknown. Using a more intact system of organotypic cultures, we show that dynamic motility of synaptic vesicle pools is also a prominent feature along developing GABAergic axons, which show very different branching and synapse distribution patterns compared with glutamatergic axons. Our current data cannot distinguish whether there was dynamic exchange of syn-GFP puncta among neighboring boutons or whether small syn-GFP packets accumulate or leave independent of their location in relation to nearby boutons. The diffraction limit of our light microscope might also lead to over-estimation of the stability of smaller vesicle pools. Importantly, we provide evidence that GABA signaling through presynaptic GABA_B_R regulates the stability of SVs, especially those small SV clusters at nascent boutons. These findings suggest a feedback mechanism whereby the strength of local GABA release itself directly regulates synaptic vesicle accumulation in the presynaptic terminal. Given the inherent constraint on bouton size (Craig et al., 2006), a release-dependent allocation of SVs could coordinate proper redistribution of synaptic weights across multiple release sites and provide a basis for the clustering of synapses with similar release probabilities onto a target, such as the perisomatic inhibitory synapses around a pyramidal cell soma and proximal dendrite.

### Presynaptic GABA_B_R regulates actin polymerization and vesicle stability in developing GABA terminals

Actin remodeling has been shown to regulate pre- and postsynaptic structural changes during synapse formation (Colicos et al., 2001; Pielage et al., 2005; Cingolani and Goda, 2008). As a stable scaffold as well as a dynamic filament in presynaptic terminals, actins maintain the stability (Sankaranarayanan et al., 2003) and regulate the motility (Kuromi and Kidokoro, 1998; Darcy et al., 2006) of synaptic vesicle pools. For example, actin turnover contributes to the sharing of vesicles between neighboring boutons (Darcy et al., 2006), which might allow individual boutons to quickly shrink or expand vesicle pools during synapse formation and plasticity. This activity-dependent adjustment of SV pools is likely achieved through signaling pathways that modulate actin polymerization. For example, BDNF-trkB signaling have been implicated in the local regulation of SV (Staras et al., 2010), and activity-induced rapid presynaptic maturation involves actin polymerization through Cdc42 (Shen et al., 2006). However, how *synaptic activity* is translated into the regulation of actin polymerization in presynaptic terminals, especially GABAergic terminals, is still not understood. Here we show that in developing GABAergic terminals, actin polymerization also influences synaptic vesicle dynamics. Importantly, we provide evidence that GABA signaling through presynaptic GABA_B_R locally modulates actin polymerization, which provides a mechanism to rapidly regulate the stability or mobility of SV pools within boutons by GABA release itself. Consistent with previous findings at developing glutamatergic synapses (Zhang and Benson, 2001), we found that small and potentially immature nascent boutons are sensitive to GABA_B_R-mediated regulation of actin polymerization. Larger and more mature boutons are more resistant to GABA transmission deficiency as well as actin depolymerization, and may have recruited additional mechanisms (i.e., synaptic adhesion molecules) to stabilize presynaptic contents.

### Presynaptic GABA_B_R regulates release-dependent maturation of GABA terminals

GABA_B_Rs located on mature glutamatergic or GABAergic axon terminals (Gonchar et al., 2001; Kruglikov and Rudy, 2008) mediate presynaptic inhibition of transmitter release (Bettler et al., 2004). GABA_B_R is also prominently expressed in postnatal developing cortex (Bettler et al., 2004), although their precise localization and function along developing GABAergic axons is not known. Our single cell deletion of GABA_B_R in basket interneurons suggests a cell autonomous action in regulating inhibitory synapse development, and the rapid effects of GABA_B_ agonists and antagonists on SV dynamics is consistent with the involvement of axonal receptors. Importantly, our focal iontophoresis directly demonstrated the highly local action of presynaptic GABA_B_R in regulating actin dynamics in developing GABAergic terminals. Together these results indicate a rapid autocrine signaling whereby GABA release feeds back to modulate presynaptic maturation. It is unknown whether presynaptic GABA_B_R at *developing* GABA terminals also mediate suppression of GABA release (Lee and Soltesz, 2011), which likely depends on the presence of appropriate downstream signaling machinery. How GABA_B_R dependent activity is translated into the regulation of actin polymerization in presynaptic terminals remains to be elucidated.

**Figure 10:**
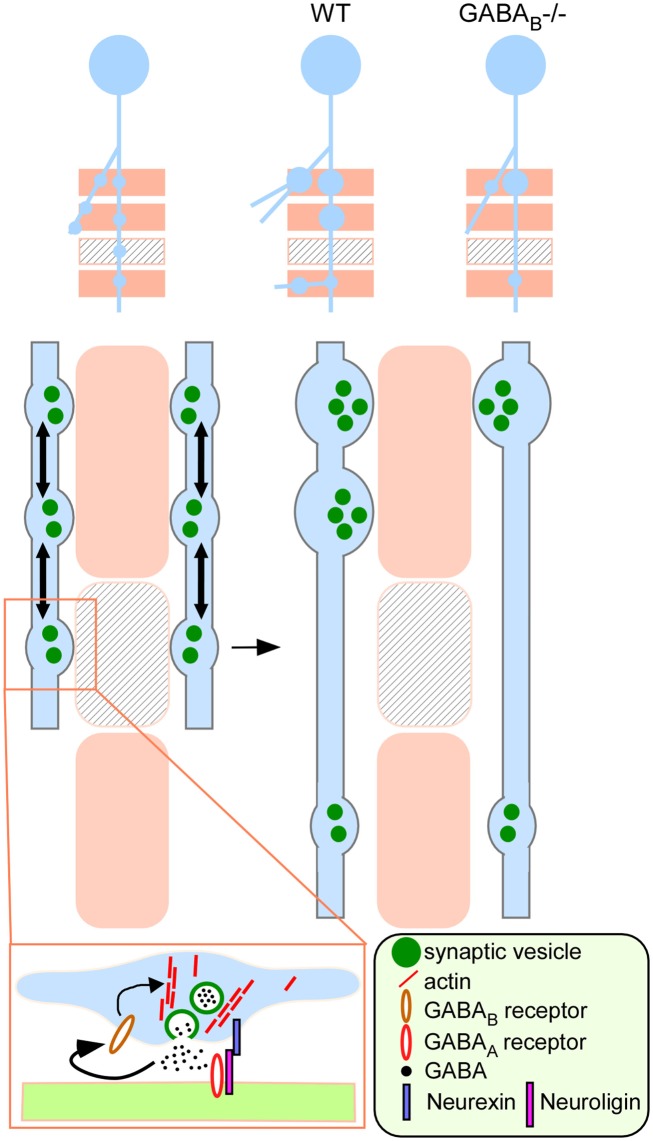
**A model on the role of presynaptic GABA_B_R in regulating the development of inhibitory synapses**. A developing GABAergic axon (light blue) explores potential synaptic targets (beige rectangles) by making transient synaptic contacts. Beige rectangles represent appropriate targets; rectangles with lines represent inappropriate targets. These transient contacts contain release machinery, such as synaptic vesicles (green filled circles) that are mobile (arrows) along the axon and mediate GABA release (boxed inset). Autocrine GABA signaling through presynaptic GABA_B_R promotes actin polymerization likely through G protein signaling and stabilizes mobile synaptic vesicles at the developing terminal. Through synaptic activity and GABA_B_R dependent recruitment and redistribution of presynaptic resource (e.g., synaptic vesicles), contacts at the inappropriate targets are eliminated whereas those at the appropriate targets are validated and strengthened (WT). In the absence of GABA_B_R (GABA_B_R*^−/−^*), nascent contacts fail to stabilize mobile synaptic vesicle pools according to GABA release, leading to reduced bouton density and aberrant distribution along axon branch.

GABA_B_Rs are G protein-coupled receptors that predominantly link to G_iα_− and G_oα_ (Bettler et al., 2004). The G_i/o_ signaling network regulates many aspects of neurite outgrowth, including axon guidance, branching, and extension (He et al., 2006). Interestingly, G_i/o_ signaling has been shown to stimulate the actin polymerizing enzyme Arp 2/3 through Cdc42-WASP (Welch and Mullins, 2002) and suppress the depolymerizing enzyme cofilin through the Rac-PAK-LIMK pathway (Manser et al., 1994; Arber et al., 1998; Yang et al., 1998; Edwards et al., 1999). These signaling pathways therefore provide a plausible mechanism by which presynaptic GABA_B_Rs regulate actin polymerization and SV pools. Similarly, the G_i/o_-coupled presynaptic M2 mAchR regulates motorneuron terminal stability at the developing neuromuscular junction (Wright et al., 2009). We have recently shown that presynaptic GABA_B_R also modulates the turnover rate of the key synaptic adhesion molecule NRX1β at developing inhibitory synapses (Fu and Huang, 2010). Therefore, this “presynaptic GABA sensor” not only regulates the stability and maturation of nascent boutons but also their adhesion to postsynaptic targets. Our studies thus begin to link GABA release, presynaptic GABA_B_R signaling, actin polymerization, regulation of synaptic vesicle pool and synaptic adhesion in the context of activity-dependent formation of inhibitory synapses (Figure 10).

Our findings raise a number of questions and unresolved issues for future studies. For example, it is not clear whether and how the dynamics and maturation status of presynaptic boutons relate to postsynaptic targets. It is possible that nascent boutons which contact inappropriate targets (e.g., mismatched cell type or subcellular compartment), although capable of transmitter release by mobile vesicles, cannot support sustained transmission and lose their SV to nearby boutons, which contact appropriate postsynaptic targets and progressively increase their vesicle pools and release strength. Testing this hypothesis would require simultaneous labeling of a basket cell axon and its appropriate (e.g., pyramidal cell somata) and inappropriate (e.g., spines) targets. Second, recent studies suggest that, in addition to presynaptic GABA_B_Rs, postsynaptic GABA_A_Rs contribute to regulating inhibitory synapse formation (Chattopadhyaya et al., 2007; Poulopoulos et al., 2009), but the underlying mechanism remains to be defined.

## Conflict of Interest Statement

The authors declare that the research was conducted in the absence of any commercial or financial relationships that could be construed as a potential conflict of interest.

## Supplementary Material

The Supplementary Material for this article can be found online at http://www.frontiersin.org/Cellular_Neuroscience/10.3389/fncel.2012.00057/abstract

Supplementary Movie S1 **Rapid short term dynamics of syn-GFP and TdTomato puncta in developing PV neuron axons**. PV neurons in cortical slice culture were transfected with *P_G67_-TdTomato* and *P_G67_-syn-GFP* at EP14. The labeled neuron was imaged with two-photon microscopy at EP18. The images were taken at 20 s interval. Image at each time point is a maximum projection of *Z*-stacks. The punctum on the right side was always present, while the one on the left briefly disappeared and reappeared. Note that a syn-GFP punctum repeatedly appeared and disappeared in the middle site of this axon segment.

Supplementary Movie S2 **Long term imaging of syn-GFP puncta in wild type PV neuron axons**. Cortical PV neurons in slice cultures from *PV-ires-Cre* mice were transfected with *LSL-syn-GFP* and *LSL-TdTomato* at EP14. The labeled neuron was imaged with two-photon microscopy at EP18. The images were taken at 1 min interval. Image at each time point is a maximum projection of *Z*-stacks. Only the syn-GFP signal is presented. While most of the syn-GFP puncta were stable, small population were dynamic.

Supplementary Movie S3 **Actin depolymerization destabilizes syn-GFP puncta in PV neurons**. Cortical PV neurons in slice cultures of *PV-ires-Cre* mice were transfected with *LSL-syn-GFP* and *LSL-cofilin(S3A)-mCherry* at EP15. The labeled neuron was imaged with two-photon microscopy at EP19. The images were taken at 1 min interval. The image at each time point is a maximum projection of *Z*-stacks. Only the syn-GFP signal is presented. Compared with control neurons (Movie S2 in Supplementary Material), Cofilin(S3A) expression resulted in many more unstable syn-GFP puncta, which appeared and disappeared on many sites along the axon.

Supplementary Movie S4 **Actin polymerization stabilizes syn-GFP puncta in PV neurons**. Cortical PV neurons in slice cultures of *PV-ires-Cre* mice were transfected with *LSL-syn-GFP* and *LSL-cofilin(S3D)-mCherry* at EP15. The labeled neuron was imaged with two-photon microscopy at EP19. The images were taken at 1 min interval. The image at each time point is a maximum projection of *Z*-stacks. Only the syn-GFP signal is presented. Compared with control neurons (Movie S2 in Supplementary Material), Cofilin(S3D) expression resulted in more stable syn-GFP puncta, which rarely disappeared.

Supplementary Movie S5 **GABA_B_R KO destabilizes syn-GFP puncta in PV neuron axons**. Cortical PV neurons in slice cultures of *GABA_B1_^flx/flx^*::*PV-ires-Cre* mice were transfected with *LSL-syn-GFP* and *LSL-mCherry* at EP15. The labeled neuron was imaged with two-photon microscopy at EP19. The images were taken at 1 min interval. The image at each time point is a maximum projection of *Z*-stacks. Only the syn-GFP signal is presented. Compared with control neurons (Movie S2 in Supplementary Material), GABA_B_R KO neurons showed more unstable syn-GFP puncta.

Supplementary Movie S6 **Cofilin(S3D) rescues unstable syn-GFP puncta in GABA_B_R KO PV neurons**. Cortical PV neurons in slice cultures of *GABA_B1_^flx/flx^*::*PV-ires-Cre* mice were transfected with *LSL-syn-GFP* and *LSL-cofilin(S3D)-mCherry* at EP15. The labeled neuron was imaged with two-photon microscopy at EP19. The images were taken at 1 min interval. The image at each time point is a maximum projection of *Z*-stacks. Only the syn-GFP signal is presented. Cofilin(S3D) overexpression resulted in the stabilization of puncta in GABA_B_R KO neurons (compare with Movie S5 in Supplementary Material).
